# Phylogeography and Population Genetics of the Kentucky Endemic Cave Beetle Genus *Neaphaenops* Jeannel (1920) (Coleoptera: Carabidae: Trechini)

**DOI:** 10.1002/ece3.74186

**Published:** 2026-09-28

**Authors:** Mahamad Sayab Miya, Elise Valkanas, T. Keith Philips

**Affiliations:** ^1^ Systematics and Evolution Laboratory, Department of Biological Sciences Western Kentucky University Bowling Green Kentucky USA; ^2^ Department of Entomology and Plant Pathology University of Arkansas Fayetteville Arkansas USA; ^3^ Center for Agricultural Data Analytics University of Arkansas Fayetteville Arkansas USA; ^4^ Department of Biology St. Mary's College of Maryland St. Mary's City Maryland USA; ^5^ Commercial Diagnostics and Bioinformatics Sarepta Therapeutics Cambridge Massachusetts USA

**Keywords:** Carabidae, CO1 gene, Mississippian Plateau, *Neaphaenops*, phylogenetics, trechines

## Abstract

Cave beetles are an excellent group for studying evolutionary history and speciation within cave habitats. However, they are underrepresented in such research, particularly in North America, a hotspot of cave biodiversity, where beetles account for a significant proportion of documented taxa. This study aims to elucidate the evolutionary history and possible connectivity or barriers to gene flow among populations of the central Kentucky endemic cave beetle, *Neaphaenops tellkampfii* (Erichson), and to test the validity of four proposed subspecies. We collected specimens from 62 caves, extracted DNA, and sequenced 1169 bp of the Cytochrome C Oxidase Subunit I (CO1) gene, resulting in 36 phylogenetically informative sites for the ingroup. Using this CO1 data, we constructed Bayesian, Maximum Likelihood, and Parsimony phylogenetic trees and also visualized the genetic structure. All the analyses illustrated two distinct southern and northern mega‐populations of this beetle, separated by the Barren River. Based on molecular evidence, here we propose a new concept that the four morphologically defined subspecies should be considered invalid. The three northern subspecies: *N. t. tellkampfii* (Erichson), *N. t. viator* Barr, and *N. t. henroti* Jeannel should be recognized as the single species *N. tellkampfii* (Erichson), while the southern subspecies *N. t. meridionalis* Barr should be raised to species level as *N. meridionalis* Barr, New Status. This study also discovered 26 new *Neaphaenops* populations. High genetic similarity was observed within each clade, potentially indicating a high level of historical or recent interconnectivity among the caves within the Mississippian Plateau that facilitates underground dispersal and gene flow. Together, our study shows that *Neaphaenops* or other cave beetles can serve as a biological model for studying speciation, gene flow, evolutionary history, and connectivity within cave systems.

## Graphical abstract

This study investigated the evolutionary history and population structure of the genus *Neaphaenops*, using CO1 gene sequencing from 62 cave populations. The phylogenetic analysis revealed two distinct mega‐populations—northern and southern—separated by the Barren River, leading to the conclusion that the four proposed subspecies are invalid. The results propose elevating the southern subspecies to species level ( *N. meridionalis* ), while the northern subspecies are unified under *N. tellkampfii* , with high genetic similarity suggesting massive interconnectivity within the Mississippian Plateau's cave system.

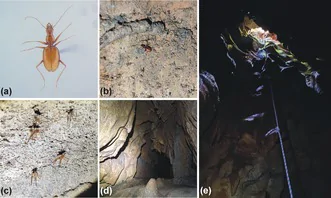

## 1 Introduction

Caves are characterized by the complete lack of light, resulting in the absence of photosynthetic primary productivity, constant temperature, and high humidity (Barr 1967; Culver and Pipan 2019). Much like island systems, they host numerous potentially isolated populations of a diverse array of specialized taxa (Barr 1968a; Culver and Pipan 2009). Cave species are hypothesized to have evolved from the independent invasion of surface ancestors into cave habitats (Hobbs III 2012; Faille et al. 2013). They are thought to have originated either from the climatic relict hypothesis and/or the adaptive shift hypothesis. The former suggests that caves acted as refugia for surface fauna in unfavorable environments, forcing them to adapt to underground habitats (Peck and Finston 1993). The latter suggests that subterranean colonization is driven by the opportunity to discover new resources and by strong natural selection that drives adaptation to this novel niche (Howarth 1987). However, while some suggest there is limited phylogenetic evidence supporting either one of these hypotheses (Ribera et al. 2010), this appears to be changing (Romero and Green 2005; Juan et al. 2010; Hart et al. 2025; Loria et al. 2022; Le Cesne et al. 2025). Regardless, the lack of gene flow with surface taxa that essentially represent the ancestral form, limited ability to disperse through underground corridors, and physical isolation are thought to have led to speciation in cave organisms, such as in beetles (Barr 1967; Porter 2007; Ribera et al. 2010; Hobbs III 2012).

Most cave organisms have generally restricted distributions, low dispersal abilities, and are often quite diverse (including cryptic taxa) (Trontelj et al. 2009; Juan et al. 2010), making them an ideal model for studying biogeography/or phylogeography and speciation (Holsinger 1991; Năstase‐Bucur et al. 2022). However, their evolutionary and speciation mechanisms are difficult to explore (Juan et al. 2010; Balart‐García et al. 2023), mainly due to the challenge of sampling (Wynne et al. 2018; Mammola et al. 2021), morphological convergence, and extinction of surface ancestors (Porter 2007; Juan and Emerson 2010). Nevertheless, recent advances in genetic techniques have provided greater insight into this field of biology (Recknagel and Trontelj 2022; Liu et al. 2023). The phylogeographic approach, which integrates genetic and geographic data, has been used to study cave faunas to shed light on biogeographic history, the relative roles of dispersal and vicariance, cryptic species diversity, colonization history, and modes of speciation (Juan et al. 2010; Năstase‐Bucur et al. 2022; von Rintelen et al. 2024). In a terrestrial cave environment, beetles often make up the most diverse insect group, “catching the eyes” of researchers for evolutionary and phylogenetic studies (Faille et al. 2015; Ribera et al. 2018; Chen et al. 2021), and are considered an excellent model group for these inquiries (Barr 1959). However, they are underrepresented in phylogenetic studies, even though they account for about 20% of all described cave‐restricted species diversity in the USA and Canada (Niemiller et al. 2019).

In eastern North America, the trechine cave beetles (Coleoptera: Carabidae: Trechinae: Trechini) include five genera: *Ameroduvalius* Valentine, *Darlingtonea* Valentine, *Neaphaenops, Nelsonites* Valentine, and *Pseudanophthalmus* Jeannel; the last genus alone constitutes more than 200 species (Barr 2004; Ober et al. 2022). The karst system in central Kentucky, home to the longest mapped cave system in the world (i.e., Mammoth Cave), is one of the hotspots for subterranean biodiversity and endemism in North America (Christman et al. 2005; Deharveng et al. 2024). One of the endemic taxa in this region is the monotypic cave beetle genus *Neaphaenops* Jeannel (Erichson 1844), which includes *N. tellkampfii* (also spelled as “*N. tellkampfi”* in some studies) with four subspecies (Barr 1979). The genus was established by Jeannel (1920) for *Anophthalmus tellkampfii* Erichson (1844). First collected in Mammoth Cave, it is also the first trechine carabid beetle described in North America (Barr 1959). *Neaphaenops tellkampfii* is also the largest (6.5–7.5 mm), most abundant, and widely distributed beetle in Mammoth Cave. Often found along silty and sandy locations, it is commonly known as the sand beetle (Barr 1962). It is highly associated with the cave cricket *Hadenoecus subterraneus* Scudder, and feeds primarily on its eggs and nymphs (Barr 1967; Norton et al. 1975). The broad distribution of this relatively vagile beetle species has been hypothesized as due to the large body size of an active forager and the highly cavernous karst limestone of the Mississippian Plateau (Barr 1967, 1979). Because cave‐adapted species can be extremely sensitive to temperature and/or humidity, most cannot survive for extended periods outside of cave habitats or submerged underwater (Barr and Peck 1965; Pallarés et al. 2019; Apostolopoulos and Philips 2022). This reduces the likelihood of above‐ground or floodwater dispersal in these groups (Barr and Peck 1965), although accidental transport by floodwater or even surface migration can occur (Barr 1959; Benito et al. 2025).

*Neaphaenops tellkampfii* has been substantially studied regarding its general biology, including food and feeding behavior and ecology (Barr 1967; Kane et al. 1975; Norton et al. 1975; Kane and Poulson 1976; Griffith and Poulson 1993). A few studies focused on body morphology (Horn 1871, 1882; Studier 1996) and brain anatomy (Packard 1888; Ghaffar et al. 1984). Barr (1959, 1962, 1966) attempted to discover new populations, while others focused on population ecology (Durant and Robert 1979; Kane and Ryan 1983) or adaptation (Culver 1987; Griffith 1991). Lastly, several studies explored genetic variability and gene flow using allozyme data (Giuseffi et al. 1978; Turanchik and Kane 1979; Caccone 1985; Kane and Brunner 1986). Unfortunately, the small sample sizes and allozyme data used in these studies, conducted before DNA sequencing technologies existed, can be quite misleading regarding population relationships.

Since the initial discovery of *N. tellkampfii* , four subspecies have been described (Jeannel 1949; Barr 1959, 1979). These are *N. t. tellkampfii* (Erichson), *N. t. viator* Barr, *N. t. henroti* Jeannel, and *N. t. meridionalis* Barr (Figure 1). Subspecies can be defined as groups within a species that are potentially interbreeding and phylogenetically distinct but reproductively compatible with other such groups (Mayr 1942; Patten and Unitt 2002). Reydon and Kunz (2021) have suggested that subspecies can exhibit differences in ecology, morphology, or genetic traits, often across geographic space, with some degree of reproductive incompatibility. The subspecies rank has historically been used by systematics to represent geographic variation within a species (Mayr 1982; Burbrink et al. 2022). Regarding *N. tellkampfii*, Philips et al. (2013) first tested the validity of the proposed subspecies using CO1 and arginine kinase sequence data from 40 taxa (including outgroups) collected across 27 caves. Based on this population‐limited study, one subspecies was valid, a second possibly so, while the other two were not.

**FIGURE 1:**
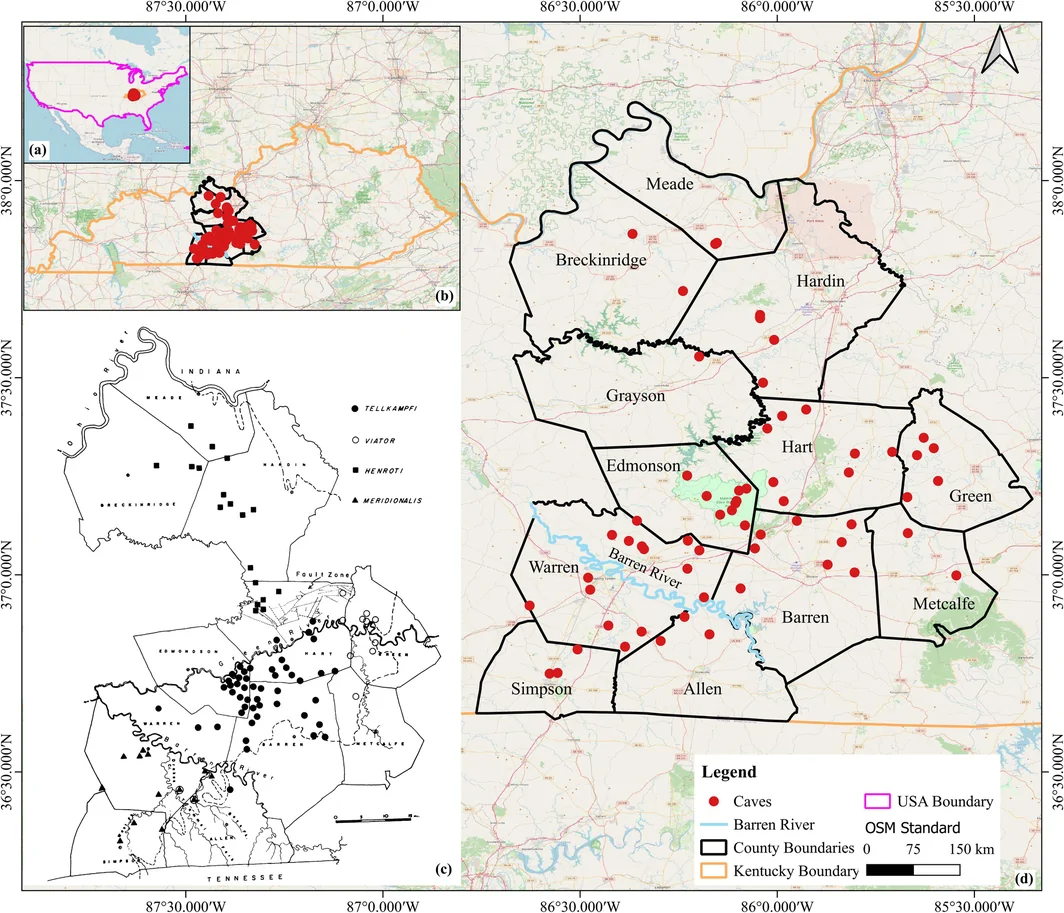
Study area map showing (a) USA boundary, (b) Kentucky boundary, and (c) county boundaries with distributions of the four subspecies in Barr (1979) (used with permission), and (d) the location of the 62 caves where *Neaphaenops tellkampfii* was collected for this study. Samples from 59 caves produced usable data. Notably, the Barren River was found to be a major barrier to gene flow in this study. The map was created in QGIS version 3.44.6.

Our study aimed to evaluate the evolutionary history of the genus *Neaphaenops* via phylogenetic analysis using DNA sequence data and, in doing so, test the hypothesis of monophyly of the proposed subspecies and identify potential barriers to gene flow or connectivity among caves or cave systems. Additionally, we discovered new populations of this beetle, further expanding the known range. Analyzing relationships among *Neaphaenops* populations provides critical insight into the evolution of this species and other cave‐dwelling organisms across the Mississippian Plateau.

## 2 Materials and Methods

### 2.1 Study Area

Specimens of *N. tellkampfii* were collected from 62 caves in central Kentucky, USA, across as much of its known distribution as possible (Figure 1). The species was previously recorded from 95 caves within this region, known as the Mississippian Plateau (Barr 1979). During field sampling, a few caves were found gated, and property owners could not be located to obtain permission to enter. Others, unfortunately, had their entrances bulldozed shut or were inaccessible due to flood debris, and in one case, piles of used tires blocked the cave entrance. Lastly, some caves could not be located, and the entrances had likely disappeared. Meanwhile, new caves were explored, and some resulted in the discovery of new populations. Sampled caves included those in Meade and Breckinridge Counties in the north, near the Ohio River, and in Simpson and Allen Counties in the south, near the Tennessee border. The northernmost cave sampled was Penitentiary Cave (37.864930° N and −86.367040° W), the southernmost was Hoy Cave (36.749620° N and −86.577280° W), the easternmost was Neal's Chapel Cave (36.998610° N and −85.545855° W), and the westernmost was Wheeler Cave (36.922070° N and −86.627990° W) (Table 1 and Figure S1). The studied caves were mostly horizontal, although a few were accessed via vertical pits or sinks.

**TABLE 1:** List of the caves sampled with the county names, number of *Neaphaenops tellkampfii* individuals (*N*) collected, cave sample code, and GenBank accession numbers for DNA (CO1) sequences.

Cave name	County name	*N*	Cave sample code	GenBank accession number
Austin Entrance*	Edmonson	3	AUST N‐1, AUST N‐2	PV639594, PV639587
Bald Knob Pit	Hart	3	BKP1, BKP2	PV639566, PV639582
Bear*	Allen	3	BEAR N‐2, BCMA1	PV639618, PV639616
Big Bat*	Breckinridge	1	BBC1	PV639581
Black Jack*	Simpson	2	BLAC N‐1, Neaph Black	PV639602, PV639603
Bland	Hardin	4	BCN1, BCN2	PV639560, PV639575
Boyce Wafer*	Warren	3	BOY N‐1, BOY N‐3	PV639619, PV639620
Brush Creek**	Green	3	BRU N‐1**, BRU N‐2**	**
Buchanan	Allen	3	BUCH N‐1, BUCH N‐2	PV639604, PV639605
Buckner Spring (Hollow)	Hart	3	BUCK N‐1, BUCK N‐2	PV639562, PV639563
Camp Branch (Gentry)**	Green	2	GCB1**, GCB2**	**
Cooch Webb	Hart	3	COWE 1, COWE 2	PV639595, PV639579
Crump (Smith Groove)	Warren	3	CRCS1**, CRCS2	PV639552
Crump*	Grayson	1	N8	PV639550
Danny Crane*	Hart	3	DCC1, DCC2	PV639583, PV639592
Devils Den	Metcalfe	2	DDC1, Neaph Devils**	PV639523
Drake‐Saddler*	Simpson	1	DSC1	PV639610
Fisher Ridge*	Hart	3	FIRI 1, FIRI 2	PV639577, PV639576
Friendship*	Warren	3	FRIE 1, FRIE 2	PV639608, PV639607
Frozen Niagra*	Edmonson	3	FROZN 1**, FROZN 2	PV639571
Glasgow*	Barren	3	GC1**, GC2	PV639520
Great Onyx	Edmonson	4	N9, GRE N‐2**	PV639573
Hanson 2*	Barren	4	HC1, HC2	PV639597, PV639572
Hoy	Simpson	3	HOYC N‐1, HOYC N‐2	PV639606, PV639609
Jack Johnson	Allen	3	JAC N‐1**, JAC N‐3	PV639617
Junior*	Warren	4	JCN1, JCN2	PV639544, PV639545
L & N Railroad	Barren	3	LNC1, LNC2	PV639574, PV639586
Lampkin Park	Warren	1	N29	PV639613
Lindy Forbes*	Green	4	LFC1, LFC2	PV639527, PV639528
Little Beauty	Edmonson	2	N7, LIBE N‐1	PV639555, PV639556
Lone Star*	Hart	3	N22, WCMA1	PV639591, PV639536
Long	Edmonson	3	LC1, LC2	PV639553, PV639584
Lost River (Big Bertha)*	Warren	3	BBE1, BBE2	PV639611, PV639614
Lutes Spring (Crump)	Hart	3	LSC2, LSC3	PV639522, PV639526
Milby	Green	3	MILB N‐1, MILB N‐2	PV639525, PV639529
Neals Chapel	Metcalfe	1	N25	PV639533
New Moo*	Warren	3	NEWM N‐1, NEWM N‐2	PV639547, PV639549
Old Forester*	Warren	3	N16, OFC2	PV639541, PV639542
Penitentiary	Breckinridge	2	PEC1, PEC2	PV639585, PV639593
Pick handle Wilmoth	Hardin	3	PWC1, PWC2	PV639564, PV639568
Pick handle Wilmoth Pit*	Hardin	3	PWP1, PWP2	PV639567, PV639570
Pit*	Barren	1	PC1	PV639559
Plano Saltpeter	Warren	4	PLSC1**, PLSC2	PV639612
Pruett Saltpeter	Warren	4	PSC2**, PSC3	PV639546
Riders Mill	Hart	3	N17, RMC1.1	PV639569, PV639561
Roaring River (Mammoth)**	Edmonson	4	ROAR N‐1**, ROAR N‐2**	**
Scott (Meade Co)	Meade	3	SCOM 1, SCOM 3	PV639521, PV639558
Scotts (Green Co)	Green	2	STS N‐1, STS N‐2	PV639524, PV639530
Sig Shackletts	Meade	1	N14	PV639557
Slick Rock	Barren	3	SRC1, SRC2	PV639534, PV639535
Smith Valley	Edmonson	2	SMIT N‐1, SMIT N‐2	PV639580, PV639598
Son of Finney*	Warren	2	SFC1, SFC2	PV639537, PV639538
Sophys*	Edmonson	3	SOPH N‐1, SOPH N‐2	PV639554, PV639565
Studebaker Sink*	Warren	3	STU N‐1, STU N‐2	PV639539, PV639540
Swinging Bridge*	Edmonson	1	SBC1	PV639551
White Onyx	Barren	4	WOC1, WOC2	PV639589, PV639578
Walnut Hill	Barren	4	WHC1, WHC2	PV639599, PV639590
Wheeler	Warren	3	WHEE 2, WHEE 3	PV639615, PV639601
White	Edmonson	3	WHI N‐1, WHI N‐2	PV639596, PV639588
Willy Crawhorn	Barren	4	WCC1, WCC2	PV639531, PV639532
Wonderland	Hardin	2	N23, WOND N‐2**	PV639600
Wright*	Warren	3	WRIC N1, WRIC N2	PV639543, PV639548

*Note:* * = new cave populations other than Barr (1979), and ** = caves or samples excluded from the analysis.

### 2.2 Beetle Collection

*Neaphaenops tellkampfii* were sampled since June 2012 with opportunistic cave visits and were extensively collected from 2023 through December 2024. The approximate cave locations were estimated in advance from the information in Barr (1979) and Harker and Barr (1980), and coordinates were also obtained from the Kentucky Speleological Survey (KSS 2019). Up to four specimens of this beetle were collected in each cave, although only a single individual was found in eight of 62 caves. Each cave collection took from 15 min to several hours, depending on the cave's accessibility and beetle abundance. *Neaphaenops tellkampfii* were found only in caves with damp areas and populations of the cave cricket *H. subterraneus* . Beetles were generally seen relatively deep inside the caves, where the temperature was cool (56°–58° Fahrenheit), and the air had high humidity level, with the latter of particular importance (Apostolopoulos and Philips 2022). Individuals were mostly observed on the ground on sandy or other substrates, sometimes under stones or other objects, and rarely on walls or ceilings. Specimens were collected by hand into a vial containing 100% ethanol and later stored at −80°C within 24 h of collection. The caves and sample details are listed in Table 1. Figure 2 shows two images of a *Neaphaenops* beetle, *Hadenoecus* cave crickets, and a representative picture of two of the sampled caves.

**FIGURE 2:**
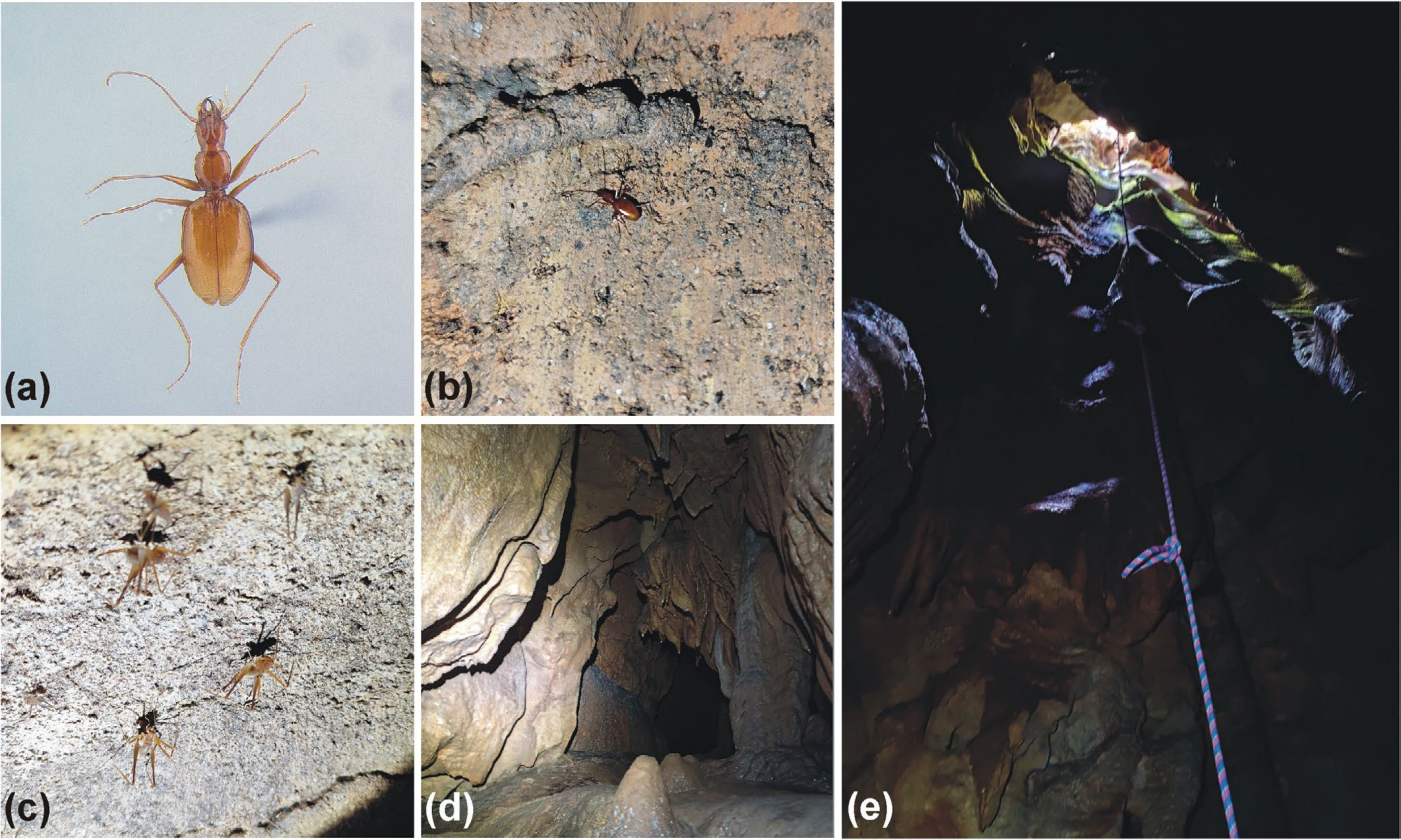
(a) Dorsal habitus of *Neaphaenops tellkampfii* , (b) an individual *N. tellkampfii* walking on the cave ground surface, (c) *Hadenoecus subterraneus* crickets on the cave ceiling, (d) horizontal and (e) vertical cave sampled during this study.

The appropriate precautions suggested by the National White‐Nose Syndrome Decontamination Protocol (older and recent v.03.2024) were followed to prevent the spread of the fungus (*Pseudogymnoascus destructans*), which has caused significant population declines in North American bat species (WNSDMWG 2024).

### 2.3 Sequencing

DNA was isolated and amplified at the Biotechnology Center, Western Kentucky University (WKU). When possible, DNA for up to four samples per cave was extracted, with the whole specimen ground up to improve yield. The isolation was performed using an E.Z.N.A. Insect DNA kit from Omega Bio‐Tek, following the given protocol. This included an initial step using a 2–3 h soaking period in a proteinase K and buffer solution, and two 75 μL elutions of DNA from the columns, resulting in a total volume of 150 μL. Each extraction typically yielded ~45–130 ng/μL of DNA. After isolation, DNA concentration and purity were quantified using a NanoDrop 2000 spectrophotometer. DNA aliquots were labeled with the concentration, date, and cave name, and stored at −80°C.

For each sample, a ~1200 bp fragment of the CO1 gene was first selected for amplification, as this rapidly evolving gene is well suited for studying taxa at the species or subspecies level, for species identification (i.e., animal DNA barcodes) or other studies of genetic diversity (Mandal et al. 2014; Lv et al. 2014; Rach et al. 2017; Pentinsaari et al. 2016; Boyd et al. 2020). The primer pairs included one standard primer (Pat) and another used in previous beetle studies (CO1F Ober), which increased the amplified fragment length by ~50%. PCR was performed (Table 2) for no more than two samples (individuals) per cave, given the relatively conserved nature of this gene, as seen in a preliminary study (Philips et al. 2013), to avoid redundancy. Two to three randomly selected PCR products from each amplification batch were visualized on an agarose gel using a FluorChem HD2 system to confirm amplification, and PCR was repeated for failed samples when known. Altogether, 115 amplified samples were sent to GENEWIZ (Azenta Life Sciences) for Sanger sequencing. Additionally, a test of the usefulness of both Wingless and Arginine Kinase nuclear gene fragments (450 and 740 bases long, respectively) from 18 caves (three south and 15 north of the Barren River) was also completed. Arginine Kinase had only three informative bases, while the Wingless gene had two; due to a lack of phylogenetic information, these two genes were excluded from the study.

**TABLE 2:** List of primers and temperature protocol used for the CO1 gene amplification of *Neaphaenops tellkampfii* .

Direction	Primer	Binding region
R	Pat	5′‐TCCAATGCACTAATCTGCCATATTA‐3′
F	CO1F Ober	5′‐CGAATAAATAATATAAGATTTTGA‐3′
Temperature gradient		
98°C—2 min initial denature		35×
98°C—10 s		
51°C—30 s		
72°C—1 min		
72°C—2 min final extension		
4°C—hold		

*Note:* The “Pat” primer is from Simon et al. (1994), and the “CO1F” Ober is newly designed (Karen Ober, unpublished).

### 2.4 Data Analysis

The CO1 sequence data obtained contained only 101 of 115 useful samples (i.e., with both forward and reverse sequencing successful). Samples from 59 of 62 caves provided data for this study, with two individuals from 42 caves and a single individual from 17 caves (Table 1). The forward and reverse sequences were merged using “*De novo* assembly,” aligned, and edited in Geneious Prime version 2025.0.3 and Geneious Pro version 5.5.9 (http://www.geneious.com). During editing, a small number of ambiguous bases were inserted where peaks in the chromatogram were distinctly higher, and all ends of the sequence reads were trimmed to equal lengths. For the outgroups, sequences of nine *Pseudanophthalmus* species with nearly similar gene fragment sizes were downloaded from NCBI GenBank (https://www.ncbi.nlm.nih.gov) (see Data Availability Statement). The final aligned and trimmed sequences contained 1169 bp for *N. tellkampfii* and outgroups, totaling 110 taxa. The genetic distance matrix for all taxa was also extracted from Geneious, and a heatmap was prepared using the pheatmap package version 1.0.13 in R version 4.5.1 (Kolde 2019; R Core Team 2025).

We used JModelTest version 2.1.6 to select the most appropriate nucleotide substitution model for phylogenetic analysis of the CO1 gene (Darriba et al. 2012). The GTR + I + G model (General Time Reversible model with Invariable sites and a Gamma distribution) was found to be the best, with the lowest Akaike Information Criterion (AIC) value (Akaike 1974). The phylogenetic tree using the maximum likelihood (ML) method was generated in MEGA version 12, with 1000 bootstrap replications (see Figure S2 for settings) (Felsenstein 1985; Nei and Kumar 2000; Kumar et al. 2024). The evolutionary rates among sites were modeled with a discrete Gamma distribution with five categories (+G, parameter = 3.7055), and 74.10% of sites were deemed evolutionarily invariant (+I). The Nearest‐Neighbor‐Interchange (NNI) heuristic method was selected for tree inference, with the default initial tree setting: the tree with the higher log‐likelihood between a Neighbor‐Joining (NJ) tree (Saitou and Nei 1987) and a Maximum Parsimony (MP) tree. The pairwise distance (*p*‐distance), defined as the number of base differences per site, was also calculated in MEGA, with 1000 bootstrap replications.

The Bayesian phylogenetic tree was created in MrBayes version 3.2.6 (Ronquist et al. 2012). The Markov Chain Monte Carlo (MCMC) chain generation was run for 4,000,000 replications, at which point the standard deviation of split frequencies reached 0.011589; values below 0.01 are generally considered adequate to end most analyses (Ronquist et al. 2020). The parameters were summarized with the command “sump,” and the trees were produced with the command “sumt” (Table S1). Parameter estimates for each run (log‐likelihood over generations) were examined for stationarity and visualized burn‐in before concluding the analyses. Trees sampled before the identified burn‐in point (after the first 25% of the runs) were discarded and excluded from summary statistics and calculations of posterior probabilities.

A parsimony tree was created using the NONA program in WinClada version 1.00.08 (Nixon 2002). In NONA, 2000 replicates were performed, holding up to 100,000 trees per step, which was the point when branch swapping was deemed unnecessary (Table S1). The number of trees, length (L), Consistency index (CI), and Retention index (RI) values were noted.

We also performed a General Mixed Yule Coalescent (GMYC) species delimitation analysis using the splits package version 1.0‐20 in R version 4.5.1 (Fujisawa and Barraclough 2013; R Core Team 2025). For this, the ingroup topology from the MrBayes consensus tree was isolated by removing outgroups. The polytomies were resolved using the “multi2di” function of the ape package version 5.8.1 (Paradis and Schliep 2019), and terminal zero‐length branches were adjusted to 1 × 10^−6^ to avoid likelihood calculation errors. An ultrametric chronogram was generated using the Non‐Negative Least Squares (NNLS) transformation using the “force.ultrametric” function of the phytools package version 2.5.2 (Revell 2012) to prevent rate‐smoothing distortions. The single‐threshold GMYC model was used to detect transitions between Yule speciation patterns and within‐species coalescent events.

Further, an unrooted split network was generated in SplitsTree version 6.0.0 based on the *p*‐distance (created a 101 × 101 distance matrix) and Neighbor Net Method (created 134 splits, cyclic) (Huson and Bryant 2024). The network helped to identify distinct genetic clusters from all *Neaphaenops* individuals and included bootstrap values. Additionally, a haplotype network was created using the minimum spanning network algorithm in PopART version 3 (Leigh and Bryant 2015). Genetic structure was partitioned into two distinct clusters (CL1 = south of the Barren River and CL2 = north of the Barren River) based on the results of all phylogenetic analyses.

## 3 Results

### 3.1 Molecular Phylogeny of *Neaphaenops tellkampfii*

Two distinct and strongly supported clades of *N. tellkampfii* were revealed across all phylogenetic analyses, corroborated by 19 bp synapomorphies using the 1169 bp fragment of the CO1 gene (Figures 3, 4, S3, S4). Twelve caves south of the Barren River formed one clade (CL1), while 47 caves north of the river formed another clade (CL2). *Pseudanopthalmus barbari* was found to be the sister taxon to *Neaphaenops*. Among all ingroup and outgroup taxa, 147 bases were informative (i.e., neither invariant nor autapomorphic), while within the ingroup, 36 bases were informative.

**FIGURE 3:**
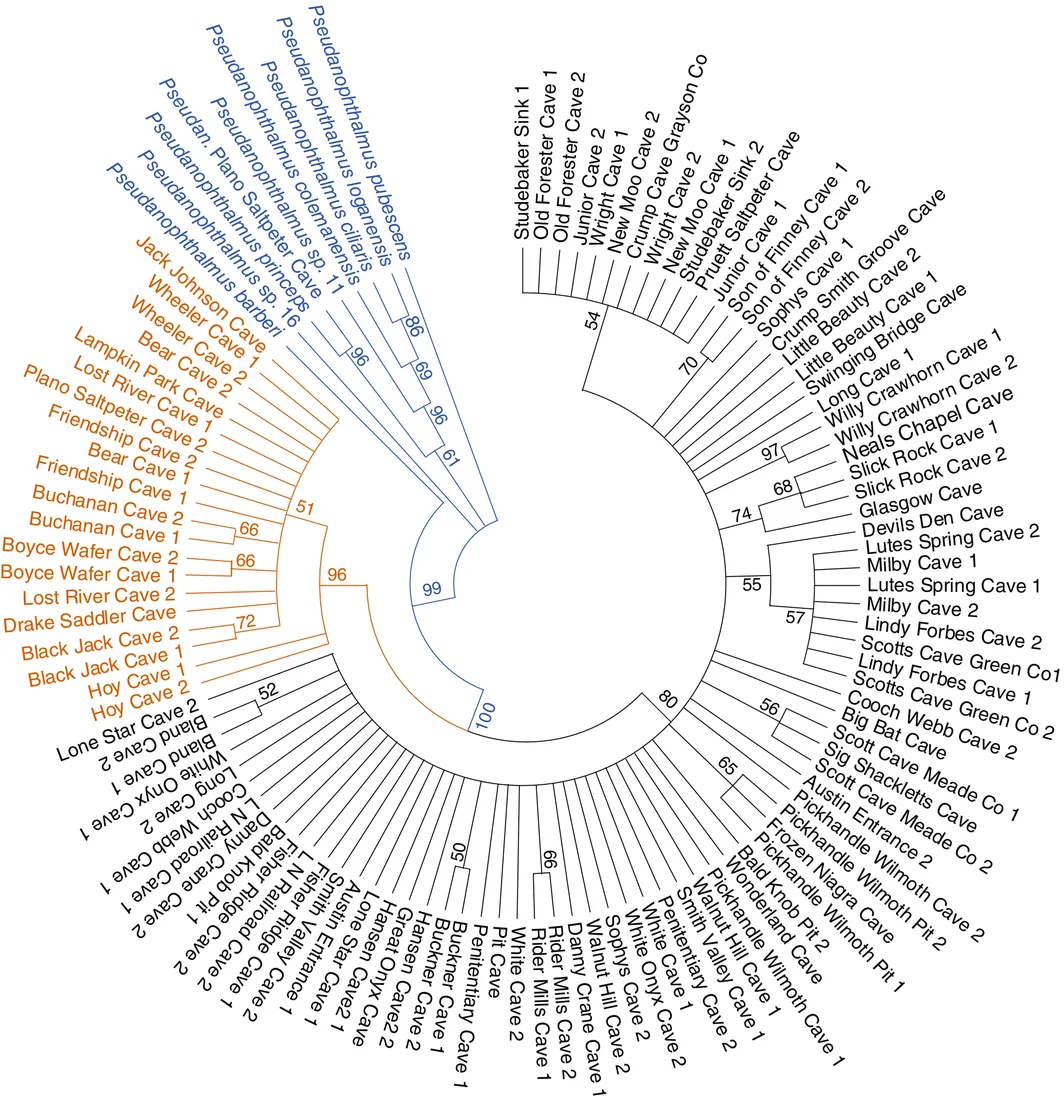
The Maximum‐likelihood phylogeny, excluding genetic distance. The tree with the highest log‐likelihood (−3151.81) is shown. Branches corresponding to partitions reproduced in < 50% of replicate trees are collapsed. The number corresponding to the branches indicates the percentage of replicate trees in which the associated taxa clustered together during 1000 replications. The cave name represents the *N. tellkampfii* individuals sampled and sequenced from each respective cave. The vermilion clade consists of the *Neaphaenops* south of the Barren River, while the dark colored clade comprises those found north of the river. The blue clade denotes the outgroup taxa.

**FIGURE 4:**
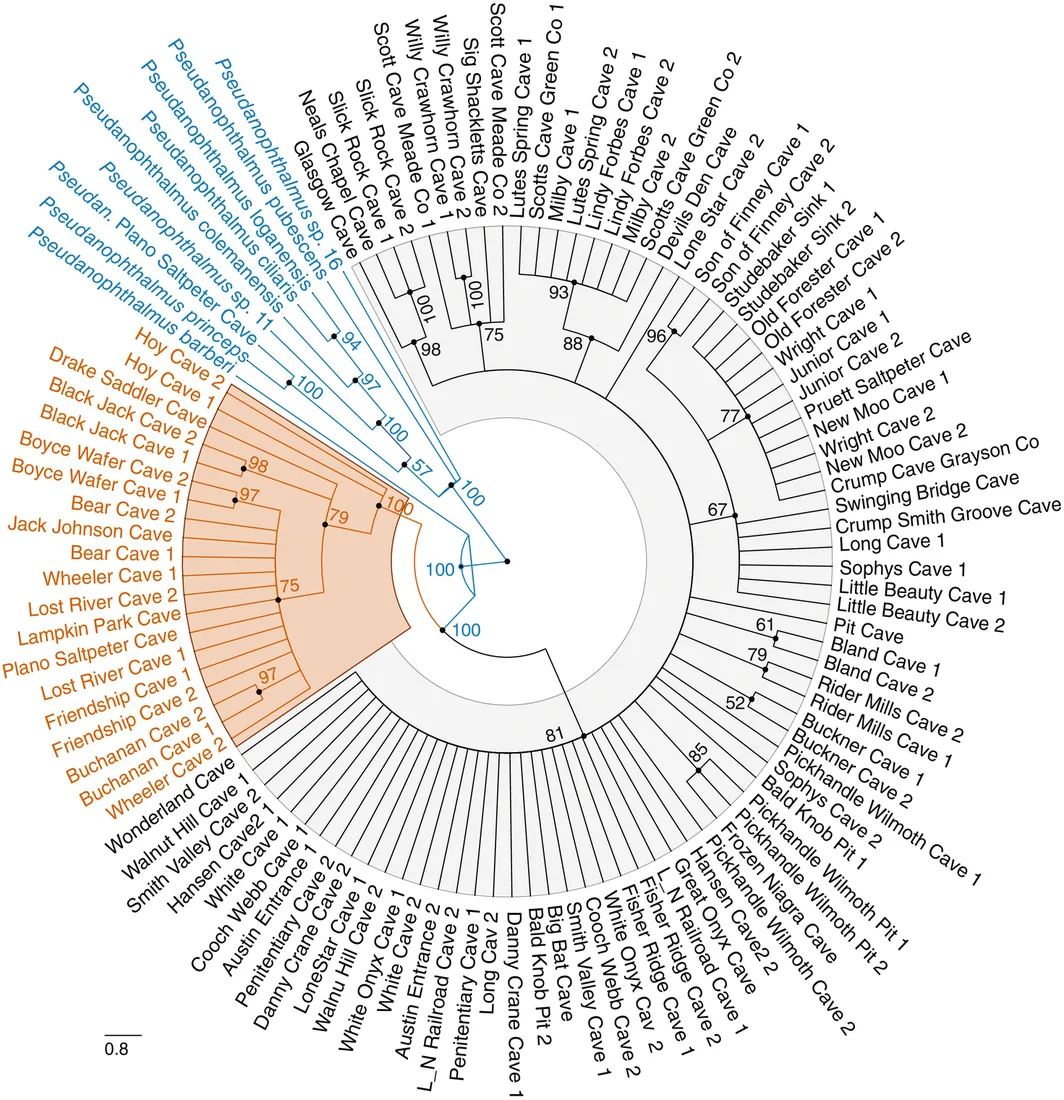
The Bayesian phylogeny with 50% Majority‐rule consensus including genetic distance. The values corresponding to the branch nodes indicate Bayesian posterior probability support. The vermilion clade consists of the *Neaphaenops* south of the Barren River, while the dark/gray shade clade comprises those found north of the river. The blue clade represents the outgroup taxa.

Phylogenetic tree generated using ML showed a strongly supported southern cluster (CL1) with a bootstrap value of 96, while the northern caves (CL2) had a bootstrap support value of 80 (Figure 3). Similarly, the Bayesian phylogenetic tree based on the 50% majority‐rule consensus revealed these same two distinct clades with high support values (CL1 = 100 and CL2 = 81) (Figure 4). The overall Bayesian tree topology aligns with the patterns observed in the ML tree. In the parsimony analysis, the strict consensus tree (L = 333, CI = 70, and RI = 91) further emphasizes this division into two mega‐populations and shows a similar loss of resolution within each clade (Figure S3) to that observed in the ML and Bayesian analyses. The majority‐rule consensus tree (L = 298, CI = 79, RI = 94) also indicated a clear phylogenetic division within *N. tellkampfii* populations, again forming two clades with strong support values; the first clade (CL1) had a bootstrap support of 100, indicating very robust support for monophyly. Conversely, the second cluster (CL2) had a bootstrap support of 85 (Figure S4). Consistent with these phylogenetic topologies, single‐threshold GMYC species delimitation further supported a two‐species model over the single‐population null hypothesis (Likelihood ratio = 52.99; *p* < 0.001). The analysis resolved two molecular clusters and two entities (95% CI: 2–2), corresponding to CL1 and CL2 (Figure S5 and Table S2).

### 3.2 Genetic Structure of *N. tellkampfii*

A split network independently confirms the results seen in the phylogenetic analyses by revealing two genetically distinct *N. tellkampfii* clusters (CL1 and CL2), with strong bootstrap support values (Figure 5). The minimum spanning haplotype network also showed the same two mega‐population clusters (Figure 6). Thirty‐one unique haplotypes were identified in the 101 *Neaphaenops* individuals sequenced; included 19 fixed nucleotide differences between CL1 and CL2, and ≤ 4 fixed differences within each of these clades. Some geographic structure is apparent with the distribution of caves and their haplotypes, as shown in Figure S6, and the haplotypes within the minimum spanning network are shown in Figure S7.

**FIGURE 5:**
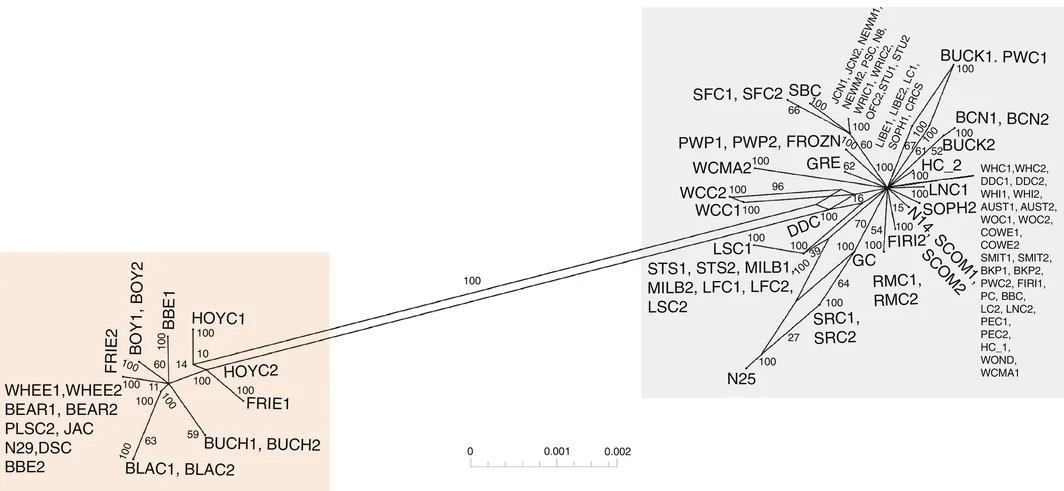
A split network created by aligning all 101 CO1 sequences using the Neighbor Net algorithm. The cave codes in vermilion shade represent the *N. tellkampfii* populations/samples south of the Barren River (CL1), while those in gray shade represent populations north of the river (CL2). Bootstrap support values are displayed for the branches, and genetic distance is indicated by the line lengths and the scale bar.

**FIGURE 6:**
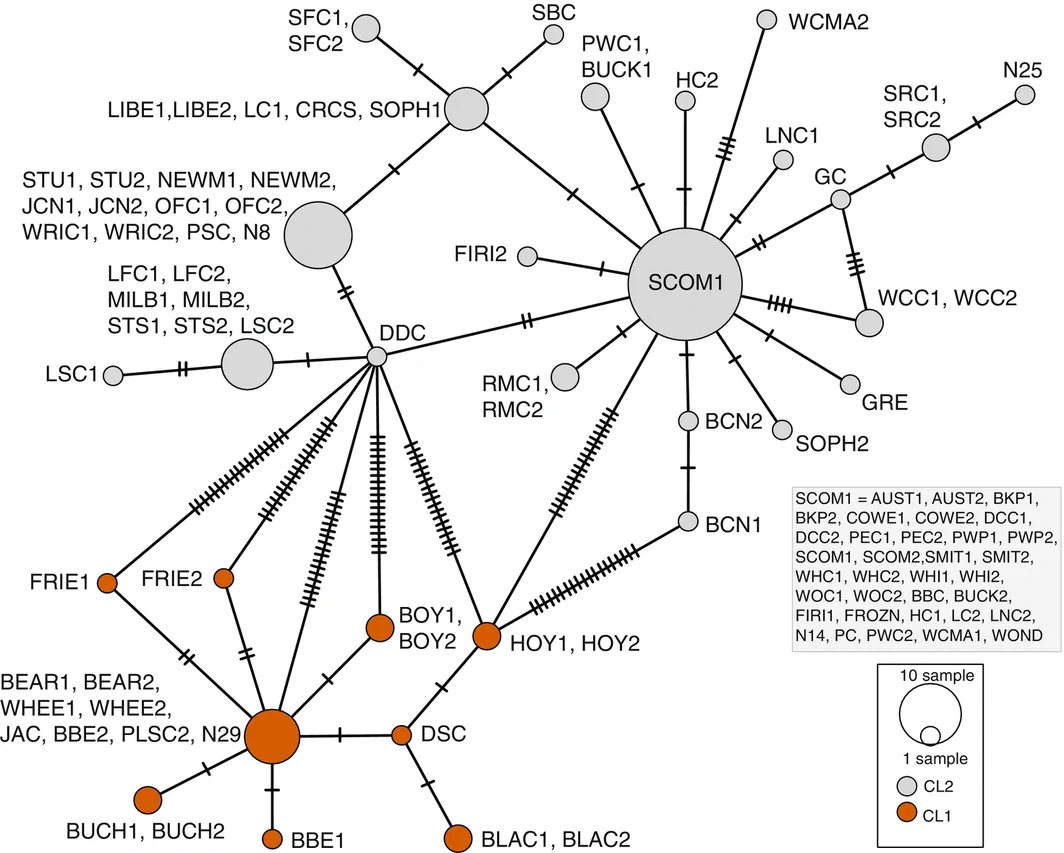
Minimum spanning haplotype network color‐coded by the two genetic clusters (CL1 = south and CL2 = north of the Barren River). Diameters represent haplotype frequencies (number of individuals contributing to that haplotype). The labels adjacent to the haplotypes represent sample codes of the corresponding caves listed in Table 1. The hatch marks along connecting lines indicate nucleotide changes between the haplotypes.

### 3.3 Genetic Similarity and Barriers to Gene Flow Between the *N. tellkampfii* Population

Based on all analyses, the Barren River is identified as a major barrier to gene flow between the two mega‐populations (Figures 1, 3, 4, 5, 6, S8). Genetic differences in the CO1 sequence between these two mega‐populations (CL1 and CL2) ranged from 1.7% to 2.7%. The genetic similarity within all sampled populations south of the river (CL1) ranged from 99.1% to 100%, while that north of the river (CL2) ranged from 99.2% to 100%. There was a relatively large genetic difference between *N. tellkampfii* and the outgroups (89.8%–92.4%). The maximum pairwise distance (*p*‐distance) for CL1 was 0.003, and for CL2 it was 0.008, while between the two *Neaphaenops* clades it was 0.022 (Table 3).

**TABLE 3:** Similarity of the CO1 gene (in percentage) and *p*‐distance between the *N. tellkampfii* populations south and north of the Barren River, as well as each compared with the *Pseudanophthalmus* outgroup.

Genetic similarity			
	*Pseudanophthalmus*	*Neaphaenops* (CL1)	*Neaphaenops* (CL2)
*Pseudanophthalmus*	90.6–100	89.8–92.1	90–92.4
*Neaphaenops* (CL1)	89.8–92.1	99.1–100	97.3–98.3
Neaphaenops (CL2)	90–92.4	97.3–98.3	99.2–100

Pairwise *p*‐distance			
	CL1	CL2	CL1–CL2
Minimum pairwise *p*‐distance	0.000	0.000	0.016
Maximum pairwise *p*‐distance	0.003	0.008	0.022

### 3.4 Historic and New Cave Records for *N. tellkampfii*

Among the total caves sampled (*n* = 62), the number of caves located in each county was as follows: five caves in Green, 14 in Warren, eight in Hart, 10 in Edmonson, eight in Barren, four in Hardin, three in Simpson, three in Allen, two in Meade, two in Breckinridge, two in Metcalfe, and one in Grayson (Table 1). New populations of *Neaphaenops* were discovered in 26 caves, and include caves from Meade (*n* = 1 cave), Hardin (*n* = 1), Grayson (*n* = 1), Hart (*n* = 3), Green (*n* = 1), Edmonson (*n* = 5), Warren (*n* = 8), Barren (*n* = 3), Allen (*n* = 1), and Simpson (*n* = 2) counties (Table 1).

## 4 Discussion

### 4.1 Molecular Phylogeny and Genetic Structure of *Neaphaenops tellkampfii*

The consistent recovery across all phylogenetic analyses of two distinct clades, one south (CL1) and the other north (CL2) of the Barren River, strongly supports a significant phylogeographic division between these *N. tellkampfii* populations. High bootstrap and posterior probability values in ML and Bayesian analyses, respectively, and substantial support in the strict consensus Parsimony tree all reinforce this strong division. Furthermore, the GMYC species delimitation results also corroborate this pattern. Similarly, the split and haplotype networks also reflect the two distinct genetic clusters, north and south of the Barren River.

Previous studies using allozymes by Giuseffi et al. (1978) and Turanchik and Kane (1979) found high genetic similarity among the *N. tellkampfii*, but these studies included samples from only five and eight caves, respectively, located in south‐central Kentucky. Kane and Brunner (1986) again used allozyme data from six enzyme systems (and an additional two scorable bands) from 18 populations, including nine from one hypothesized subspecies and two or three for the others. In contrast to our results, they found monophyletic support using genetic distance in a UPGMA analysis for two of the four proposed subspecies, although these supported clades were based on only three populations each. Two primary points can be made about the use of allozymes compared to our study, where we utilized nearly 1200 bp of the CO1 gene. First, allozymes have relatively low resolution for evolutionary history because they are solely products of gene expression (Simpson 2010). Furthermore, only eight protein bands used by Kane and Brunner (1986) likely provided very low genome coverage and failed to accurately capture the genetic diversity and population structure of *Neaphaenops*. The CO1 sequence fragment has limitations as a barcode for diverse phylogenetic studies at higher taxonomic levels; however, it serves as strong evidence for closely related populations/or species, as in this case. However, other genetic markers, such as 16S and 28S, could be used if the CO1 does not produce reliable results (Lv et al. 2014) because the CO1 gene may not always be amplified by PCR in some taxa (Chen et al. 2012).

Our study found a relatively low genetic similarity between and a high genetic similarity within each of the two major clades (CL1 and CL2) discovered. For congeneric species, a minimum interspecific genetic difference of ≥ 2% can be used as the threshold (Zhang and Bu 2022) and has been used in other insect taxonomic studies (Sharkey et al. 2021); our study meets this criterion. However, more than 30 concepts have been applied to delimit species, with their limitations, such as reproductive isolation, common origin (monophyly), morphological/genetic cohesion, or phenotypic similarity (Karbstein et al. 2024). Morphology is the most commonly used criterion for species delimitation (Dayrat 2005) but may not accurately distinguish cryptic species (Pérez‐Delgado et al. 2022). The genetic analysis has recently been used to enhance species delimitation (Dietz et al. 2023) and is used in this study.

The present genetic analysis strongly rejects the hypothesis of *N. tellkampfii* being divided into four subspecies (Barr 1979). Barr (1979), in his study conducted before the advent of DNA data, documented subtle morphological differences among his defined subspecific taxa, separated them in a key, and listed these differences in a table. Notably, he excluded characteristics of the two hypothesized hybrids and wrote the following: “Intergradation occurs in geographically intermediate populations between (a) *t. tellkampfii* and *t. meridionalis* and (b) *t. tellkampfii* and *t. viator*. Hybridization, if it has occurred in some form, is certainly secondary in the first case and possibly also in the second, where intergradation is broader and more gradual. In contrast, gene flow between *t. henroti* and either of the subspecies to the south is probably severely restricted by a stratigraphic and structural barrier, a heavily faulted ridge with considerable thicknesses of sandstone which transversely crosses Hart County (Barr 1968b; 1979).” Barr (1979) stated that the hybrid *N. t. tellkamfii* × *N. t. meridionalis* is present in Friendship Cave (Warren County) and Bear Cave (Allen County), but he did not specifically mention any cave where the second hybrid is present. This study found no evidence of hybridization. The discordance between morphology and genetics has been noted in many cave taxa (Herman et al. 2018).

To summarize, our study supports treating populations south of the Barren River as a single well‐supported monophyletic clade, in which most populations were previously assigned to *N. t. meridionalis*. All other caves north of the Barren River were assigned to one of the other three subspecies: *N. t. viator, N. t. henroti*, and *N. t. tellkampfii* (Barr 1979). Further, a stratigraphic and structural barrier, if one exists, apparently has not been present long enough to create a genetically distinct northern population named *N. t. henroti*. Therefore, the subspecies concept is rejected; all three northern subspecies (*N. t. tellkampfii* (Erichson), *N. t. viator* Barr, and *N. t. henroti* Jeannel) should be recognized as the single species *N. tellkampfii* (Erichson). The southern subspecies *N. t. meridionalis* Barr is raised herein to species level as *N. meridionalis* Barr, New Status.

The eastern North American trechines are assumed to have an archaic relationship with *Trechoblemus*, a flying beetle of Eurasian origin that may have crossed through northern routes during the Eocene into North America, successfully establishing itself in caves but becoming extinct on the surface (Valentine 1952). Jeannel (1928) demonstrated the apparent morphological similarity of *Neaphaenops* and *Pseudanophthalmus* with *Trechoblemus*. The closest known relative of *Pseudanophthalmus*, *sensu lato*, may be *Duvaliopsis* Jeannel, which Barr (1967, 1985b) believed had colonized North American caves during the Pleistocene interglacial. Benito et al. (2025) concluded that *Trechoblemus* (a descendant of *Trechus*) colonized North American caves 11.5 MYA (Miocene), giving rise to *Pseudanophthalmus*, from which various lineages further evolved into the other four eastern North American cave genera. The ancestors of troglobites likely entered caves initially as troglophiles, with gene flow between epigean and hypogean populations. The extinction of epigean populations due to climatic events, particularly during the Pleistocene, may have led to geographic and genetic isolation within caves (Barr 1967). Moreover, *Neaphaenops* also shows convergence in morphology and life‐history patterns with *D. kentuckensis* (a non‐overlapping eastern Kentucky species), including body size, food preference, and habitat selection (Norton et al. 1975).

In this study, *Neaphaenops* and its sister clade of *P. barbari* were consistently supported across all analyses. However, Benito et al. (2025), in a broader study of eastern North American trechine cave beetles, showed that *N. tellkampfii* and *P. troglodytes* (not included in this study) are sister taxa that separated ~5 MYA (Pleistocene), and both are sister to *D. kentuckensis* ; this larger clade in turn shares common ancestry with *P. caecus* that diverged about ~7 MYA (Late Miocene). In contrast, based on morphology and geographic considerations, Barr (1979) suggested close ancestry of *N. tellkampfii* with *P. pubescens* (Horn) and *P. princeps* Barr, a hypothesis that is not supported.

### 4.2 Barriers to Gene Flow Between the *Neaphaenops* and Other Cave Trechine Populations

Many trechine species are confined to a single or interconnected drainage basin (Krekeler 1959; Kane et al. 1992). Trechines are regularly found near the water in cave streams, which may frequently rise and flood some caves, and these beetles should be somewhat tolerant of transport by water (Krekeler 1959). Dispersal may occasionally occur when floods wash beetles out of caves via small surface streams, and some individuals may manage to crawl into crevices in the limestone along the stream banks, providing access to the underground cave system (Barr and Peck 1965). Barr (1985b) also concluded that there is a relationship between the barrier potential of watercourses via the meander frequency, suggesting that streams or rivers become barriers if meander frequency is low (< 1.0 meander/km). Although this physical aspect was not evaluated, this might explain why the Barren River is a barrier to dispersal and gene flow, in stark contrast to the potentially more serpentine Green River. Regarding the latter, *Neaphaenops* dispersal across this river appears to have regularly occurred based on the evidence herein and from several *Pseudanophthalmus* species that can be found in caves on either side as well.

One can further hypothesize that along the Barren River, there may also be fewer karst openings in the limestone (largely due to the presence of non‐karst sandstone) compared to that substrate along the Green River. Notably, this river also divides two species of *Pseudanophthalmus*, *P. menetriesi* (Motschulsky) and *P. transfluvialis* Barr (Barr 1985a). Other streams and rivers within the *Neaphaenops* distribution do not appear to be hindering gene flow. Barr (1979) hypothesized that small streams, as well as relatively large rivers, do not limit the dispersal of trechines when there are numerous caves or karst openings on both banks. Larger rivers, though, are almost always considered barriers to terrestrial troglobites (Barr and Holsinger 1985), as one would expect. The northern border of *N. tellkampfii* is near the Ohio River, and no populations are known to occur north of this major barrier in Indiana. The western range of this species is limited by non‐cavernous rocks of the Western Kentucky Coal Basin, and the southern and eastern range extent is limited by the contact between St. Louis and Warsaw limestones (Barr 1967), and moreover, especially further east, by the absence of *Hadenoecus* cricket populations (Barr 1979).

Both proposed species of *Neaphaenops* have wide distributions, which may be facilitated by the high vagility of this relatively large and mobile species and the openness of solutional networks in the greatly cavernous Mississippian limestones within its range (Barr 1967, 1979). The distribution of *Neaphaenops* populations is approximately 15–30 km wide and 175 km long (Barr 1967). The findings from the present study confirm that the caves within the study area are interconnected to some degree, facilitating gene flow among *Neaphaenops* populations, with the notable exception of the Barren River barrier. Alternatively, relatively recent historical connections may also be factors in similar CO1 sequences among populations. Caccone (1985), using allozyme data, also found high gene flow between the population of *N. tellkampfii* relative to other arthropod lineages (i.e., *Hadenoecus* crickets and *Ptomaphagus* leiodid beetles) of central Kentucky. Ober et al. (2022) also suggested that the interior plateau has more continuous karst and possible connectivity. Recent evidence of a troglobiont cave spider ( *Phanetta subterranea* ) distributed across a wide range in North America, yet showing low genetic diversity, also raises questions about connectivity between the cave systems present in a much larger area of eastern North America (Kennedy et al. 2024).

### 4.3 Historic and New Cave Records for *Neaphaenops*

This study explored 36 caves listed in Barr (1979) with an additional 26 caves sampled in this study; samples were collected from 12 counties, with new populations discovered in 10 of these. Perhaps surprisingly and for unclear reasons, populations of *Neaphaenops* were not found in several caves with previous records, such as Hanson (Barren County), Glass (Breckinridge), Old Smoky (Simpson), and Moats (Warren). Furthermore, entrances to some caves, including Bennett (Simpson County), Tyman (Barren), Ronalds (Hart), Belt (Breckinridge), Slates (Simpson), and Bypass (Warren), were found to now be closed by debris mostly driven by flood water. Other caves, like Brown (Barren) and Salt Peter (Green), had high water levels that prevented entry during an attempted visit, while others were private commercial sites, including Hidden River Cave (Hart) and Diamond Caverns (Barren) that were not sampled. Brushy Knob (Barren) and Thornhill Caves (Breckinridge), with the latter gated, are on private property, and landowners could not be located. Blue Level Cave (Warren) could not be found, and we suspect the entrance has disappeared. Parker Cave was close to Walnut Hill Cave (both in Barren County) and almost certainly connected; therefore, a sample from the former was deemed unnecessary. In an effort to locate the northernmost populations (Breckenridge County), the Milan Sink system and Bandy Cave were explored, but only *Pseudanophthalmus* taxa were found.

## 5 Conclusion

This phylogeographic study, based on nearly 1200 bp of the CO1 gene, showed a significant phylogenetic division, with two distinct clades of *Neaphaenops* corresponding to two mega‐populations, one each north and south of the Barren River. We propose a new concept that the clades should be referred to as two separate species, *N. tellkampfii* (Erichson) in the north and *N. meridionalis* Barr, New Status in the south. The high genetic similarity within the caves both south and north of the Green River likely indicates strong interconnectivity (at least up to relatively recent times), enabling gene flow within each region of the large karst known as the Mississippian Plateau. The discovery of new populations from previously unreported caves further expands the known distribution of *Neaphaenops* , perhaps highlighting the need for continued exploration of unsampled caves and conservation efforts. Due to the wide distributions of these two *Neaphaenops* species, both could be considered at low risk of extinction, although cave systems are generally quite vulnerable to disturbance. Future research should examine how natural disturbances, such as flooding, and anthropogenic influences, such as cave visit or pollution, influence the behavior and dispersal of this species and other troglobionts. Lastly, this species is an efficient model for assessing connectivity among caves within its range and, importantly, may reflect gene flow patterns in many other cave organisms in this region.

## Author Contributions

**Mahamad Sayab Miya:** data curation (equal), formal analysis (equal), funding acquisition (equal), investigation (equal), software (supporting), validation (equal), visualization (equal), writing – original draft (equal), writing – review and editing (equal). **Elise Valkanas:** data curation (equal), formal analysis (supporting), investigation (equal), writing – original draft (supporting), writing – review and editing (equal). **T. Keith Philips:** conceptualization (lead), data curation (equal), formal analysis (equal), funding acquisition (equal), investigation (equal), methodology (lead), project administration (lead), resources (lead), software (lead), supervision (lead), validation (equal), visualization (equal), writing – original draft (equal), writing – review and editing (equal).

## Funding

This work was funded by a Graduate Student Research Grant, The Graduate School, Western Kentucky University (WKU) (Grant Index #221707). This work also received Open Access Publishing Fund, administered through the University of Arkansas Libraries.

## Conflicts of Interest

The authors declare no conflicts of interest.

## Supporting information


**Figure S1:** Map showing the distribution of caves with their names from where *N. tellkampfii* was sampled. The map was created in QGIS version 3.44.6.
**Figure S2:** The setting used for the maximum likelihood phylogenetic tree construction.
**Figure S3:** Strict consensus of 100 trees from parsimony analysis (L = 333, CI = 70, and RI = 91) that show two Neaphaenops clades, one south (vermilion shade) and the other north (gray shade) of the Barren River. The cave name represents the *N. tellkampfii* individuals sampled and sequenced from each respective cave. The blue‐colored clade denotes the outgroup taxa.
**Figure S4:** The majority rule (50%) tree from parsimony analysis (L = 298, CI = 79, and RI = 94). The bootstrap values are 100 for the vermilion‐shade clade and 85 for the gray‐shade clade. The cave name represents the *N. tellkampfii* individuals sampled and sequenced from each respective cave. The blue‐colored clade denotes the outgroup taxa.
**Figure S5:** Diagnostic plots from the GMYC species delimitation analysis for *N. tellkampfii* . (a) Pruned ultrametric chronogram, with black ancestral branches indicating macroevolutionary speciation and red terminal branches highlighting the two delimited clusters. (b) Lineage‐through‐time (LTT) plot, where the vertical red line marks the maximum likelihood threshold time (−0.00167), capturing the sharp transition from inter‐species speciation to rapid intra‐species population coalescence. (c) Profile likelihood curve showing a single, sharp maximum likelihood peak supporting a two‐species model.
**Figure S6:** Distribution map of samples used for haplotype network. Each circle represents a cave location, with smaller circles indicating a single individual sampled and larger, divided circles indicating two individuals sampled. Each color indicates a unique haplotype, of which 31 were discovered.
**Figure S7:** Minimum spanning network of 31 CO1 haplotypes color‐coded by haplotype number. Diameters represent haplotype frequencies (number of individuals contributing to that haplotype). The labels adjacent to the haplotypes represent sample codes of the corresponding caves listed in Table 1. The hatch marks along connecting lines indicate nucleotide changes between the haplotypes.
**Figure S8:** Heatmap showing the genetic distance (%) between the CO1 gene of *N. tellkampfii* . The codes represent the caves listed in Table 1.
**Table S1:** The command used to execute the phylogenetic tree in MrBayes and NONA.
**Table S2:** Summary of the General Mixed Yule Coalescent (GMYC) single‐threshold species delimitation analysis.

## Data Availability

The CO1 sequence data have been deposited in GenBank. Accession numbers—SUB15316095: PV639520–PV639620. Data released upon publication. The scripts and other data for the analyses presented are available in the following GitHub repository: https://github.com/sayab321/Neaphaenops.
