# Supplementary material for: Phylogeography and Population Genetics of the Kentucky Endemic Cave Beetle Genus Neaphaenops Jeannel (1920) (Coleoptera: Carabidae: Trechini)
Source: Ecol Evol. 2026 Sep 28;16(10):e74186. doi: 10.1002/ece3.74186 (PMC13619357; doi:10.1002/ece3.74186)
Supplement: Supplementary file 1 [file ECE3-16-e74186-s001.docx]

**Phylogeography and population genetics of the Kentucky endemic cave beetle genus *Neaphaenops* Jeannel (1920) (Coleoptera: Carabidae: Trechini)**

Mahamad Sayab Miya, Elise Valkanas, T. Keith Philips

**Figure S1**. Map showing the distribution of caves with their names from where N. tellkampfii was sampled. The map was created in QGIS version 3.44.6.

**Figure S2**. The setting used for the maximum likelihood phylogenetic tree construction.

**Figure S3**. Strict consensus of 100 trees from parsimony analysis (L = 333, CI = 70, and RI = 91) that show two *Neaphaenops* clades, one south (vermilion shade) and the other north (gray shade) of the Barren River. The cave name represents the *N. tellkampfii* individuals sampled and sequenced from each respective cave. The blue-colored clade denotes the outgroup taxa.

**Figure S4**. The majority rule (50%) tree from parsimony analysis (L = 298, CI = 79, and RI = 94). The bootstrap values are 100 for the vermilion-shade clade and 85 for the gray-shade clade. The cave name represents the *N. tellkampfii* individuals sampled and sequenced from each respective cave. The blue-colored clade denotes the outgroup taxa.

**Figure S5.** Diagnostic plots from the GMYC species delimitation analysis for N. tellkampfii. **a)** Pruned ultrametric chronogram, with black ancestral branches indicating macroevolutionary speciation and red terminal branches highlighting the two delimited clusters. **b)** Lineage-through-time (LTT) plot, where the vertical red line marks the maximum likelihood threshold time (-0.00167), capturing the sharp transition from inter-species speciation to rapid intra-species population coalescence. **c)** Profile likelihood curve showing a single, sharp maximum likelihood peak supporting a two-species model.

**Figure S6**. Distribution map of samples used for haplotype network. Each circle represents a cave location, with smaller circles indicating a single individual sampled and larger, divided circles indicating two individuals sampled. Each color indicates a unique haplotype, of which 31 were discovered.

**Figure S7**. Minimum spanning network of 31 CO1 haplotypes color-coded by haplotype number. Diameters represent haplotype frequencies (number of individuals contributing to that haplotype). The labels adjacent to the haplotypes represent sample codes of the corresponding caves listed in Table 1. The hatch marks along connecting lines indicate nucleotide changes between the haplotypes.

**Figure S8.** Heatmap showing the genetic distance (%) between the CO1 gene of *N. tellkampfii*. The codes represent the caves listed in Table 1.

**Table S1**. The command used to execute the phylogenetic tree in MrBayes and NONA.

| Steps | Commands |
| --- | --- |
|  | MrBayes |
| 1 | log start filename = xxx.txt (xxx is the name of my file) |
| 2 | Execute “xxx.nex” |
| 3 | lset nst = 6 rates = invgamma |
| 4 | mcmc ngen = 1,000,00 (starting), added the replication value until the split frequency reached less than 0.01. |
| 5 | sump (summarize parameters) |
| 6 | sumpt (summarize tree) |
|  | NONA |
| 1 | Hold100000 |
| 2 | mult*1000+1000 |
| 3 | rs0 |
| 4 | sv*xxx.tre |

**Table S2**. Summary of the General Mixed Yule Coalescent (GMYC) single-threshold species delimitation analysis.

| Parameter | Value |
| --- | --- |
| Model Type | Single-threshold |
| Likelihood of Null Model | 971.2024 |
| Maximum Likelihood of GMYC Model | 997.6949 |
| Likelihood Ratio (LR) | 52.9851 |
| Likelihood Ratio Test (*p*-value) | 3.12 × 10^-12^ |
| Number of ML Clusters | 2 |
| Confidence Interval (Clusters) | 2–2 |
| Number of ML Entities | 2 |
| Confidence Interval (Entities) | 2–2 |
| Threshold Time | -0.001667 |

*Note: significance level: p < 0.05*
